# Humic Acid–Functionalized Starch Gel Coatings for Controlled-Release Urea Fertilizer via Wurster Fluidized-Bed System

**DOI:** 10.3390/gels12040281

**Published:** 2026-03-27

**Authors:** Babar Azeem, KuZilati KuShaari, Muhammad Umair Shahid, Muhammad Zubair Shahid, Abdul Basit

**Affiliations:** 1 Department of Chemical Engineering, College of Engineering, Imam Mohammad Ibn Saud Islamic University (IMSIU), Riyadh 11564, Saudi Arabia; 2 Chemical Engineering Department, Prince Mohammad Bin Fahd University, Al Khobar 34754, Saudi Arabia; kkushaari@pmu.edu.sa; 3 Centre of Innovative Nanostructures & Nanodevices (COINN), Universiti Teknologi PETRONAS, Bandar Seri Iskandar 32610, Perak, Malaysia; 4 Interdisciplinary Research Center for Hydrogen Technologies and Carbon Management, King Fahad University of Petroleum & Minerals, Dharhan 31216, Saudi Arabia; 5 Department of Chemical Engineering and Technology, University of Gujrat, Gujrat 50700, Pakistan; dr.abdulbasit@uog.edu.pk

**Keywords:** humic acid, starch gel coating, Wurster fluidized-bed, biodegradability, nutrient release kinetics, carnauba wax, controlled-release urea

## Abstract

Sustainable fertilizer technologies are essential to address nutrient losses, environmental pollution, and inefficiencies associated with conventional urea application. In this study, humic acid–functionalized starch (St–HA) gel coatings were developed and optimized via a Wurster fluidized-bed system to produce controlled-release urea granules, with an additional carnauba wax outer layer to further extend nutrient release duration. The coating formulation was synthesized through in situ crosslinking of tapioca starch with humic acid using N,N′-methylenebisacrylamide and potassium persulfate, yielding a cohesive film. A central composite rotatable design (CCRD) was employed to investigate the influence of atomizing air pressure, fluidizing air flow rate, fluidized-bed temperature, and spray rate on coating performance. Comprehensive characterization; including FTIR, XRD, rheological analysis, thermogravimetric studies, water retention, biodegradability, and surface abrasion, confirmed chemical crosslinking, structural stability, and mechanical robustness of the coatings. Nitrogen release analysis in both water and soil demonstrated a substantial extension of release longevity from less than 2 days (uncoated) to 18–20 days for St–HA-coated urea, and up to 28 days with the additional wax coating. Coated granules exhibited low abrasion (8–24%), high water-retention capacity, and 68% biodegradation in 60 days, ensuring environmental compatibility. The findings establish St–HA/wax hybrid coatings as a viable, eco-friendly strategy for controlled-release fertilizers, integrating renewable feedstocks with scalable industrial processing for precision nutrient management.

## 1. Introduction

The increasing global demand for agricultural productivity, coupled with the urgent need to minimize environmental degradation, has intensified interest in advanced fertilizer delivery systems [1]. Conventional nitrogen fertilizers, particularly urea, suffer from substantial nutrient losses through volatilization, leaching, and denitrification, resulting in low nitrogen-use efficiency (NUE) and significant environmental consequences such as eutrophication and greenhouse gas emissions [2]. Controlled-release fertilizers (CRFs) have emerged as a promising solution by enabling gradual nutrient release in synchrony with plant demand, thereby improving NUE, reducing application frequency, and mitigating environmental losses. However, many commercial CRFs rely on petroleum-derived, non-biodegradable polymer coatings, which persist in soils and pose long-term ecological risks [3].

Biodegradable gel-coatings based on renewable polysaccharides offer a sustainable alternative, combining environmental safety with functional performance [4]. Starch, an abundant, inexpensive, and film-forming polysaccharide, has attracted considerable attention for CRF applications [5]. Its biodegradability, hydrophilicity, and chemical modifiability allow tailoring of release profiles [6]. Yet, native starch coatings often exhibit inadequate mechanical strength, poor water resistance, and limited durability under soil conditions, necessitating chemical or physical modifications to improve performance [7].

Humic acid (HA), a naturally occurring macromolecule derived from the decomposition of organic matter, offers multiple agronomic benefits beyond serving as a coating modifier [8]. Its incorporation into fertilizer coatings can enhance soil structure, stimulate microbial activity, improve nutrient chelation, and promote root development [9]. Moreover, its aromatic and phenolic structures can interact with polysaccharides via hydrogen bonding and covalent crosslinking, potentially improving coating strength, hydrophobicity, and resistance to premature dissolution [8]. Despite these advantages, systematic integration of humic acid into starch-based coatings via controlled crosslinking, and the evaluation of such coatings for CRF production in industrially relevant fluidized-bed systems, remains scarcely reported.

The Wurster fluidized-bed coating process is widely recognized for producing uniform, defect-free polymer films on granular substrates through precise control of spray, drying, and fluidization conditions [10]. While this technique has been extensively applied for pharmaceutical coatings and CRF production, its application to functionalized starch–humic acid systems, is underexplored. Additionally, the influence of processing parameters on coating quality, surface abrasion resistance, and nutrient release behavior has not been comprehensively investigated using statistically robust approaches such as central composite rotatable design (CCRD).

The present study addresses these gaps by developing a crosslinked starch–humic acid coating formulation and applying it to urea granules via Wurster fluidized-bed coating. The coating formulation was optimized for rheological, thermal, and mechanical properties, and an additional biodegradable carnauba wax sealing layer was introduced to further extend nitrogen release duration. The effects of key process parameters; atomizing air pressure, fluidizing gas flow rate, and spray rate; on coating quality and performance were systematically evaluated using CCRD. Comprehensive characterization, including Fourier-transform infrared spectroscopy (FTIR), X-ray diffraction (XRD), thermogravimetric analysis (TGA/DSC), surface abrasion testing, biodegradability assessment, water retention evaluation, and nutrient release kinetics in water and soil, was performed. The outcomes provide new insights into the design of bioactive CRFs that combine enhanced nitrogen-release longevity with soil health benefits, offering a scalable pathway toward sustainable fertilization practices.

## 2. Results and Discussion

### 2.1. Characteristics of Coating Formulation

#### 2.1.1. Rheological Analysis

**Figure 1:**
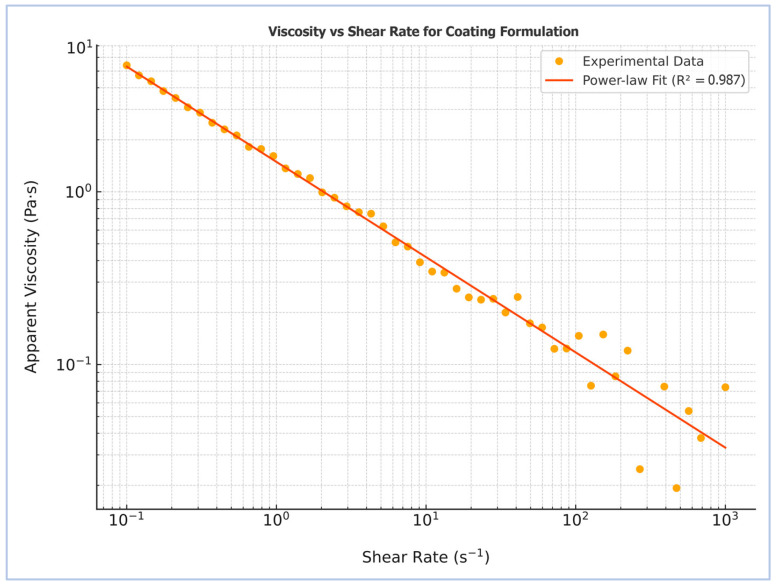
Rheological profile of the starch–humic acid-based coating formulation after crosslinking.

The rheological profile of the starch–humic acid (St–HA) coating solution was evaluated to determine its suitability for pneumatic atomization in a Wurster fluidized-bed system. As shown in Figure 1, the apparent viscosity of the coating formulation exhibited a pronounced dependence on shear rate, decreasing consistently from ~4.5 Pa·s at low shear (0.1 s^−1^) to ~0.05 Pa·s at high shear rates (1000 s^−1^). This behavior is characteristic of pseudoplastic or shear-thinning fluids, where the alignment and disentanglement of polymer chains under increasing shear reduces internal resistance to flow [11].

The experimental data were fitted to the Power law model, yielding a high coefficient of determination (R^2^ = 0.987), confirming excellent agreement with the theoretical model for non-Newtonian fluids. The calculated flow behavior further confirms the pseudoplastic nature of the formulation. This rheological profile is advantageous for Wurster-based spray coating applications, as the high viscosity at rest ensures stability and minimal settling, while the low viscosity at high shear facilitates fine atomization and uniform film deposition over urea granules.

The observed shear-thinning behavior is attributed to the presence of flexible polysaccharide chains from starch, further entangled and partially crosslinked with humic acid via hydrogen bonding and covalent bridges. The humic acid likely contributes additional viscosity-modulating effects through its polyfunctional structure and polyelectrolyte nature. This viscoelastic response supports not only effective processing but also the formation of conformal coatings capable of maintaining mechanical integrity and resisting premature dissolution upon soil contact.

#### 2.1.2. Fourier-Transform Infrared Spectroscopy (FT-IR)

**Figure 2:**
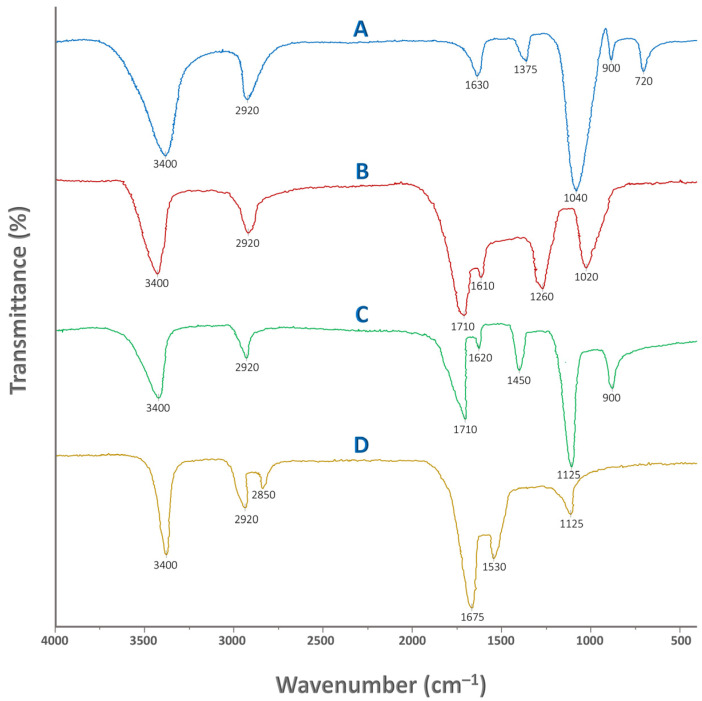
FTIR spectra of: (A) pure Tapioca starch, (B) pure humic acid, (C) physically mixed starch and humic acid, and (D) crosslinked starch and humic acid.

Figure 2 illustrates the FTIR spectra of pure Tapioca starch, humic acid, physical mixture of the aforementioned, and crosslinked St–HA formulation. FTIR analysis was carried out to confirm the successful incorporation and interaction of humic acid and the crosslinking agent within the starch-based coating matrix. This characterization technique provides insight into the presence and alteration of functional groups, enabling us to distinguish between mere physical mixing and chemical crosslinking. By comparing the spectra of pure starch, pure humic acid, their physical mixture, and the final crosslinked formulation, we were able to detect structural changes and infer possible bond formations.

In particular, FTIR helps verify whether the radical-mediated grafting and crosslinking reactions have occurred, as intended, during the coating synthesis. It also supports the evaluation of chemical stability, interaction mechanisms, and the presence of reactive groups that may influence the nutrient release behavior. The detailed peak assignments for all four cases are summarized in Table 1, providing a consolidated interpretation of the spectral data. Thus, FTIR serves as a critical tool for validating the functionalization strategy employed in designing the smart, humic-acid–enhanced controlled-release fertilizer system.

The crosslinking and functionalization reaction of the starch–humic acid (St–HA) formulation proceeds via a free-radical graft polymerization mechanism initiated by the redox pair of potassium persulfate (K_2_S_2_O_8_) and sodium metabisulfite (Na_2_S_2_O_5_). The mechanism involves the following key steps:

(I) The redox pair generates sulfate radicals ($SO_{4}^{•-}$) and other reactive species in aqueous medium (Initiation):

$$K_{2}S_{2}O_{8}+{Na}_{2}S_{2}O_{5}\to 2SO_{4}^{•-}+other\;species$$

(II) The generated radicals abstract hydrogen atoms from hydroxyl groups of starch and carboxyl/phenolic groups of humic acid, forming macroradicals (Radical Activation of Starch/Humic Acid):

$$St-OH+SO_{4}^{•-}\to St-O•+HSO_{4}^{-}$$

$$HA-COOH\;or-OH+SO_{4}^{•-}\to HA•+HSO_{4}^{-}$$

(III) The vinyl groups in N,N′-methylenebisacrylamide (MBAA) undergo free-radical polymerization and simultaneously crosslink with the activated sites on both starch and humic acid (Graft Copolymerization with MBAA):

$$St•+CH_{2}=CH-CONH-CH_{2}-NHCO-CH=CH_{2}\to Crosslinked\;St-MBAA-St$$

St•+MBAA+HA•→St−MBAA−HA

(IV) Continued radical propagation leads to the formation of a 3D crosslinked network of starch chains, humic acid molecules, and MBAA bridges. The humic acid may be physically embedded and/or covalently bonded, depending on the availability of reactive sites (Network Formation).

#### 2.1.3. Thermogravimetric Analysis (TGA)

**Figure 3:**
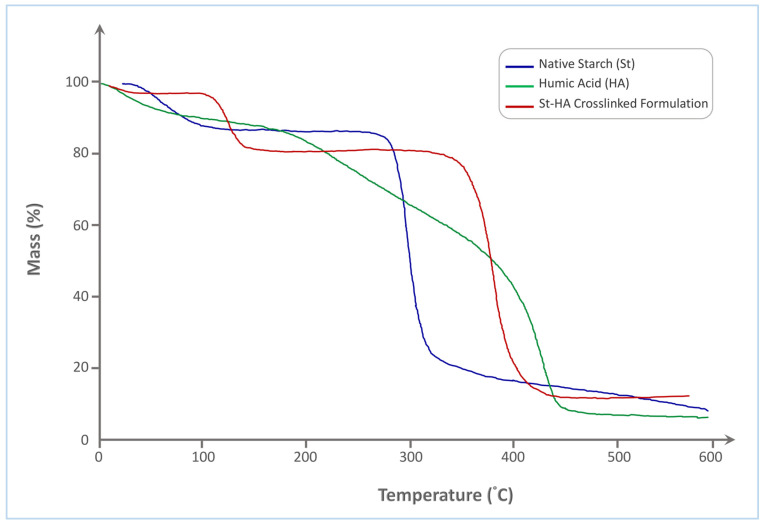
TGA thermogram of Tapioca starch, humic acid, and crosslinked St–HA.

The thermogravimetric analysis (TGA) curves of native starch (St), humic acid (HA), and the St–HA crosslinked formulation (Figure 3) reveal distinct thermal degradation profiles, reflecting the differences in their structural compositions and thermal stabilities.

Native starch exhibits a typical two-step degradation pattern. The initial weight loss, occurring between 50 and 120 °C, is attributed to the evaporation of physically adsorbed and bound water. The primary thermal decomposition of starch occurs sharply within the range of 290–340 °C, which corresponds to the depolymerization of the starch backbone and the cleavage of glycosidic linkages. This results in rapid mass loss, with a final residue of approximately 15%, indicating significant volatilization of organic matter.

In contrast, humic acid undergoes a more gradual and broader thermal decomposition. The initial mass loss also begins around 50–120 °C, due to water loss, followed by a broad degradation phase extending from 180 °C to 460 °C. This extended degradation range is indicative of the complex molecular structure of HA, comprising a mixture of aromatic, aliphatic, and functionalized carbon species. The thermal breakdown includes the sequential loss of carboxylic, phenolic, and alkyl functionalities. The final char residue is around 12%, slightly lower than that of native starch, reflecting its relatively lower carbonization potential.

The St–HA crosslinked formulation demonstrates a significantly improved thermal stability compared to both individual components. After initial dehydration up to 120 °C, a minor mass loss occurs in the 200–300 °C range, likely corresponding to the decomposition of labile HA fragments or non-crosslinked moieties. The major decomposition step of the crosslinked material, however, shifts to a higher temperature range of 330–420 °C, which clearly suggests enhanced thermal resistance due to covalent and hydrogen bonding interactions between starch hydroxyls and HA functional groups. This delay in degradation indicates successful formation of a crosslinked network that impedes thermal scission. Moreover, the final char residue is increased to ~20%, which is consistent with the formation of a more carbon-rich, thermally stable structure.

The shift in the main decomposition range to higher temperatures and the increase in residual mass in the St–HA formulation support the conclusion that chemical crosslinking between starch and humic acid enhances the thermal durability of the coating matrix. These improvements in thermal performance are critical for controlled-release applications where the coating must withstand processing and environmental conditions without premature breakdown.

#### 2.1.4. X-Ray Diffraction

X-ray diffraction analysis was employed to investigate the crystallographic features and structural modifications associated with the crosslinking of native starch (St) with HA. This characterization is essential in the context of urea coating development, as the degree of crystallinity directly influences key functional properties such as water uptake, barrier performance, and nutrient release kinetics.

**Figure 4:**
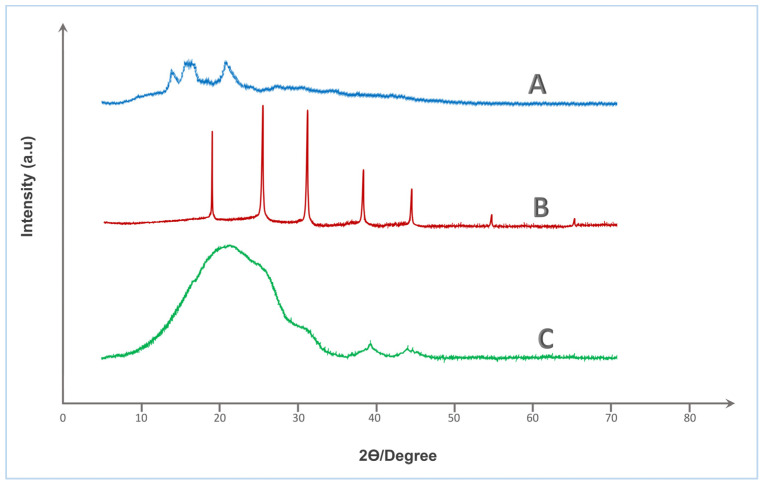
XRD spectra of (A) Tapioca starch, (B) Humic acid, and (C) Crosslinked St–HA formulation.

The XRD spectrum of native starch (spectrum A—Figure 4) displays distinct broad peaks at 2θ ≈ 15.0°, 17.1°, 18.0°, and 23.1°, which are characteristic of the A-type semi-crystalline pattern commonly associated with cereal starches. This partial crystallinity is attributed to the ordered arrangements of amylopectin chains within the starch granules. Such crystalline zones typically enhance water resistance but are susceptible to disruption upon chemical modification.

In contrast, pure humic acid (spectrum B—Figure 4) exhibits several sharp and intense peaks centered at 2θ ≈ 19.8°, 22.5°, 26.6°, 29.6°, 32.3°, 36.0°, 39.5°, 47.2°, 54.8°, and 60.0°, reflecting a relatively crystalline nature, which likely arises from inorganic mineral residues or structured aromatic domains within the humic matrix. While humic substances are generally regarded as amorphous, the presence of residual ash and metal chelates may result in diffraction peaks of crystalline origin.

The most striking transformation is observed in the XRD profile of the St–HA crosslinked formulation (spectrum C—Figure 4), which presents a dominant broad halo centered around 2θ ≈ 20.6–21.2°, accompanied by a diffuse shoulder near 40–42°, with the disappearance of all sharp reflections. This pronounced amorphization confirms the disruption of native crystalline domains of starch and the suppression of mineral crystallinity from humic acid. The observed peak broadening and decreased intensity are indicative of increased structural disorder and reduced long-range molecular order, resulting from extensive crosslinking interactions such as hydrogen bonding, esterification, and ionic complexation between hydroxyl groups of starch and functional moieties (e.g., carboxyl and phenolic groups) of humic acid.

This transition from semi-crystalline to predominantly amorphous structure is highly desirable for urea coating applications, as amorphous matrices generally offer improved film-forming ability, greater flexibility, and more homogeneous diffusion pathways for nutrient release control. The amorphous character also implies higher molecular mobility and swelling capacity, which can be fine-tuned via crosslink density to engineer targeted release profiles. Therefore, the XRD analysis validates the successful structural integration of starch and humic acid into a single-phase amorphous matrix, well-suited for developing sustainable, and functionally efficient controlled-release fertilizer coatings.

#### 2.1.5. Biodegradability Analysis of Formulation Film in Soil

**Figure 5:**
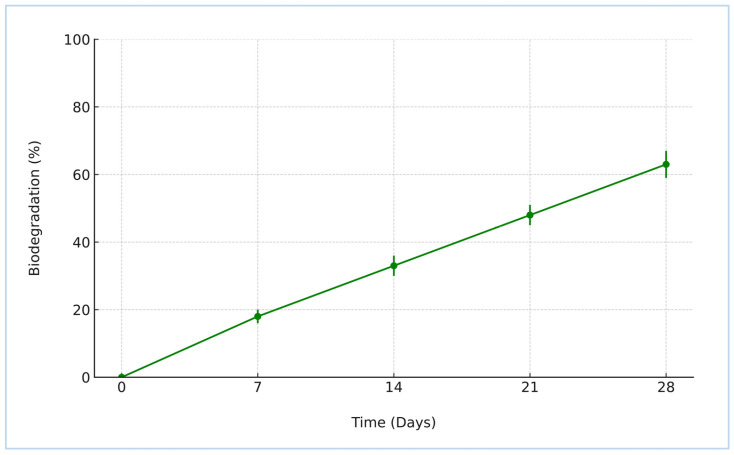
Biodegradability of crosslinked St–HA films (error bars are based on SD).

The biodegradation behavior of the synthesized crosslinked starch–humic acid (St–HA) films was investigated under simulated aerobic soil conditions over a 28-day period, and the results are depicted in Figure 5. The data show a continuous and appreciable weight loss of the film samples, increasing from 18.2 ± 1.3% on Day 7 to 63.2 ± 3.5% by Day 28. This progressive degradation trend reflects the formulation’s susceptibility to microbial and enzymatic attack under natural soil burial conditions.

The observed biodegradation kinetics can be attributed to the inherent biodegradability of both starch and humic acid, coupled with the tailored crosslinking that preserved the essential hydrolysable bonds within the polymer matrix [8]. Although crosslinking improves the mechanical stability and water resistance of the coating, it does not hinder the microbial accessibility of glycosidic linkages in starch or carboxylic and phenolic functionalities in humic acid. Over time, microbial enzymes—particularly amylases and oxidative enzymes—break down the St–HA matrix into smaller, environmentally benign byproducts such as glucose, humic residues, and low-molecular-weight organic acids [9].

This trend strongly supports the eco-compatibility of the developed coating system. The film’s steady degradation under soil conditions confirms its non-persistent and non-toxic nature, aligning well with sustainable agricultural practices. Importantly, the final biodegradation percentage (above 60% at 28 days) meets the minimum thresholds stipulated by standard protocols for claiming biodegradability in soil.

Hence, the St–HA formulation exhibits promising potential as a partially biodegradable, environmentally safe coating material for controlled-release fertilizers, mitigating long-term soil accumulation and aligning with circular bioeconomy principles.

### 2.2. Characterization of St–HA/Urea Coated Granules

**Figure 6:**
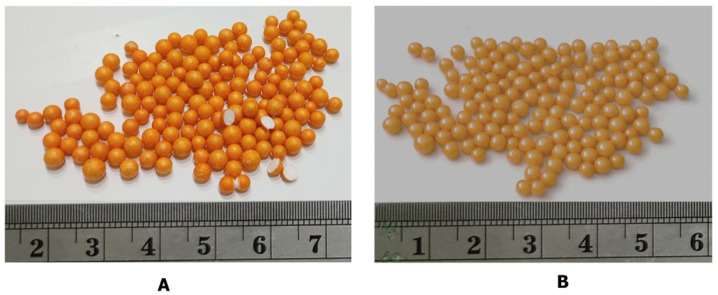
Urea granules coated with (**A**) crosslinked St–HA and (**B**) crosslinked St–HA/wax.

Figure 6 shows urea granules coated with the starch–humic acid (St–HA) formulation and the same granules further coated with an external wax layer. The wax layer not only modifies the surface color and sheen but also contributes to smoother granule morphology and occasional adhesion between particles, indicative of enhanced surface sealing and reduced surface roughness.

#### 2.2.1. Nutrient Release

**Figure 7:**
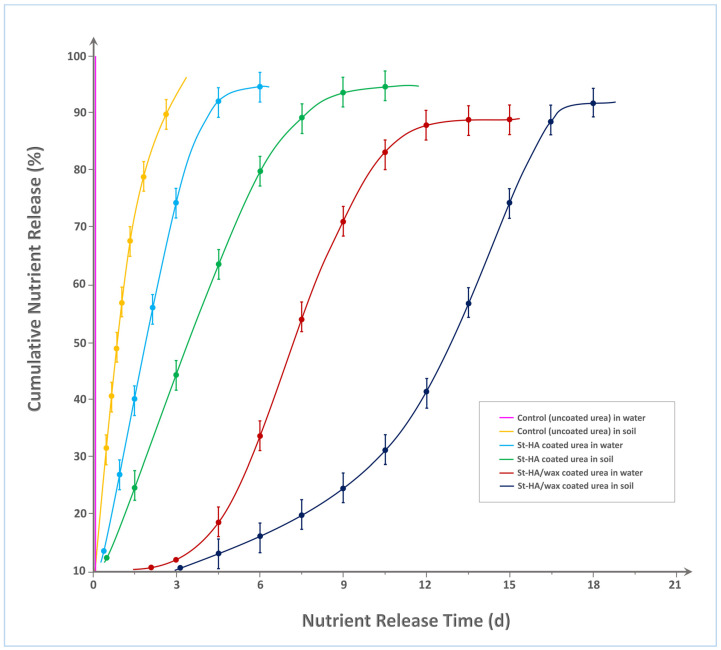
Nutrient-release characteristics of various types of controlled-release fertilizers.

Figure 7 illustrates the cumulative nitrogen release profiles of various urea formulations—uncoated (control), St–HA coated, and St–HA/wax dual-coated—under aqueous and soil conditions over 20 days. As expected, the control urea granules in water demonstrated an almost instantaneous release, reaching ≈100% nutrient dissolution within 2 h, confirming their high solubility and lack of any diffusion barrier. In soil, the control release profile was only marginally delayed, attaining ≈95% release within 3 days, likely due to slower water penetration and limited mobility in the soil matrix.

The St–HA coated urea formulation exhibited a significant improvement in release retardation. In water, the nutrient release curve showed a gradual rise, reaching 90% cumulative release around Day 7. This retardation can be attributed to the hydrocolloid matrix of crosslinked starch and humic acid, which acts as a diffusion barrier, slowing water ingress and urea efflux. In soil, the same formulation extended the release period further, attaining 90% release around Day 10, due to the additional resistance posed by soil porosity, reduced wetting, and lower microbial activity compared to aqueous systems.

Most notably, the St–HA/wax dual-coated urea demonstrated the longest nutrient release duration among all tested formulations. In water, the presence of the external carnauba wax coating significantly delayed urea dissolution, with only ≈90% nitrogen release observed at Day 13. In soil, the release profile extended even further, approaching 90% around Day 18. This extended longevity is attributed to the combined diffusion resistance offered by both the hydrophilic (St–HA) and hydrophobic (wax) layers. The wax coating, being water-repellent and biodegradable over a longer timescale, provided an additional protective shell that slowed down both water penetration and urea migration. This dual-barrier system created a synergistic retardation effect, effectively tailoring the release kinetics to span nearly three weeks.

The progressive shift in release curves to the right (from control to St–HA to St–HA/wax) confirms the strong correlation between coating complexity and nutrient release longevity. These findings validate the design rationale of using a hydrophilic-hydrophobic composite coating strategy for achieving controlled and sustained nutrient delivery, particularly suitable for minimizing nitrogen loss and optimizing uptake in agronomic applications. For comparative analysis, Table 2 indicates nutrient release from various modified-starch-based formulations/coated granules.

#### 2.2.2. Kinetics of Nutrient Release

**Figure 8:**
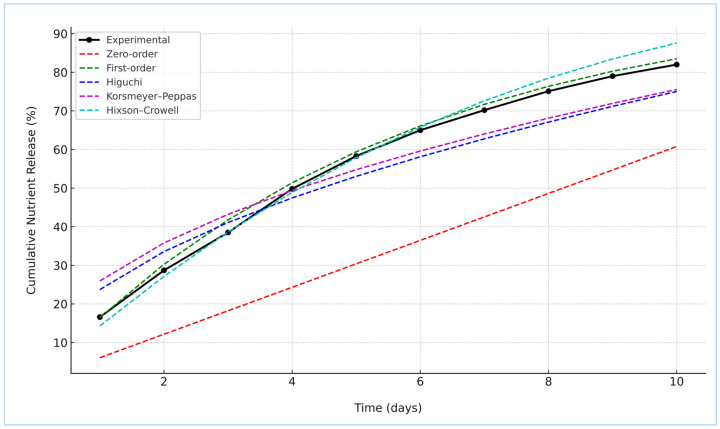
Kinetic models fitting curves with nutrient release data.

To elucidate the underlying release mechanism of nitrogen from the St–HA/wax-coated urea granules, the cumulative release data were fitted to five classical kinetic models: Zero-order, First-order, Higuchi, Korsmeyer–Peppas, and Hixson–Crowell [22]. The experimental profile and model fittings are illustrated in Figure 8. Among the tested models, the First-order kinetic model demonstrated the best agreement with the experimental data, suggesting that the nutrient release was primarily concentration-dependent. This finding indicates a diffusion-controlled release mechanism where the rate decreases with diminishing nitrogen content inside the coated granules.

The Hixson–Crowell model also showed a strong fit, implying that changes in the surface area and particle diameter during dissolution might play a secondary role. The relatively good fit of the Korsmeyer–Peppas model with an “*n*” exponent (Table 3) between 0.45 and 0.89 confirmed the anomalous (non-Fickian) transport behavior, reflecting a dual mechanism of diffusion and coating matrix relaxation or erosion. On the other hand, the Higuchi model, while still yielding a moderate fit, assumes ideal matrix geometry and may not fully represent the complexities of the composite St–HA/wax barrier. The Zero-order model, which represents constant release over time, exhibited the poorest correlation, as expected in biodegradable polymeric systems where the coating integrity and thickness progressively evolve during immersion. These results are in agreement with the studies reported by Li et al. [4], Saini et al. [23], and Sarhan et al. [24].

The kinetic modeling reinforces the effectiveness of the dual-layered coating system in modulating nutrient release. The first-order behavior aligns with our coating strategy where humic acid–functionalized starch forms the primary diffusion-limiting matrix, and the outer carnauba wax layer retards water ingress, collectively extending the nutrient release period [8,9]. These mechanistic insights are crucial for tailoring future formulations to meet crop-specific fertilization requirements and for achieving synchronization between nutrient availability and plant uptake.

#### 2.2.3. Water Retention of St–HA/Urea Granules

**Figure 9:**
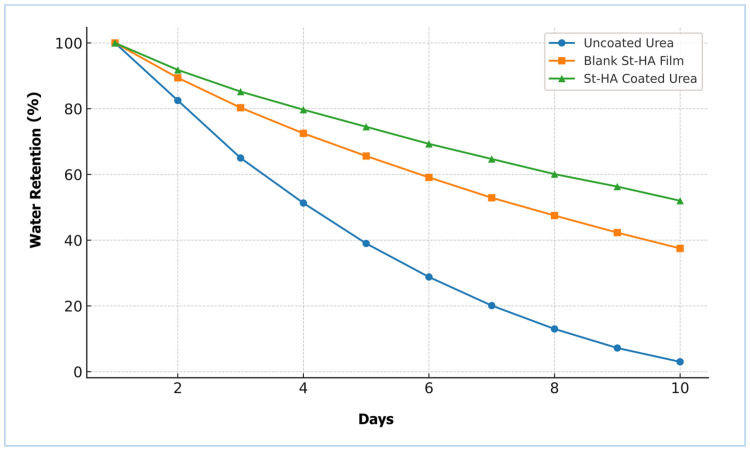
Water retention of pristine urea, St–HA film, and St–HA coated urea granules.

The water retention capacity (WRC) of the different formulations was evaluated over a 10-day period, and the results are presented in Figure 9. Uncoated urea exhibited a rapid decline in water retention, reaching nearly 3% by day 10, indicating poor moisture retention. In contrast, both the blank St–HA film and the St–HA coated urea formulations showed significantly improved water retention profiles.

The St–HA coated urea retained approximately 52% of water on day 10, compared to 37% for the blank St–HA film. This enhancement in water-holding ability can be attributed to the hydrophilic nature of starch and humic acid, and more importantly, their crosslinked matrix which acts as a hydrogel-like barrier [25]. The improved water-holding ability of the St–HA coating plays a dual role: it maintains localized moisture around the fertilizer granules and regulates the dissolution rate of urea, both of which are crucial for prolonged nutrient availability in soil.

These findings align with the reported literature where biopolymer–humic acid hybrid matrices have been shown to enhance the water retention behavior in soil-amended systems [8,9]. The significantly higher WRC of the St–HA coated urea confirms the functional advantage of the crosslinked formulation in mitigating leaching and ensuring sustained hydration—essential for arid and semi-arid agricultural conditions.

Thus, the water retention study not only supports the efficacy of the coating formulation in moisture management but also underpins its potential for improving fertilizer use efficiency under water-stressed conditions.

#### 2.2.4. Quantification of Surface Abrasion

**Figure 10:**
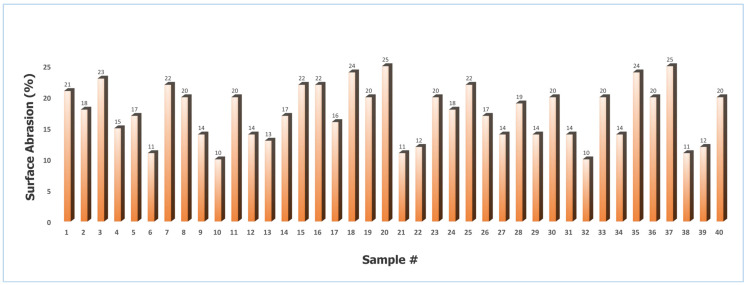
Surface abrasion of St–HA coated urea granules (Sample # stands for sample number).

Figure 10 illustrates the surface abrasion values for the coated granules ranged from 10% to 25%, with a mean of 17.7% and a standard deviation of 4.17% (CV = 23.56%). This level of variability indicates that while most batches exhibited acceptable mechanical stability, certain samples were more susceptible to wear under shear stress. The maximum abrasion value of 25% reflects significant coating deterioration, which can be attributed to specific processing conditions during the coating stage.

In particular, a high spray rate likely resulted in the deposition of a frail and mechanically weak coating layer, which lacked sufficient cohesion and adhesion to withstand repeated particle–particle collisions [26]. Similarly, excessively high coating temperatures promoted premature solvent evaporation, leading to elutriation and the formation of a thin, highly heterogeneous coating film [27]. Such non-uniform structures are inherently more prone to chipping and surface damage during handling [28]. On the other end of the spectrum, excessively low temperatures increased the viscosity of the coating solution, impairing its ability to spread evenly over the granule surface. This caused the development of fluffy, porous, and heterogeneous coatings that exhibited poor abrasion resistance [29,30].

The lowest recorded abrasion value (10%) corresponded to conditions that promoted optimal coating uniformity, adequate film thickness, and strong adhesion—factors that collectively improved mechanical durability. The results confirm that the surface abrasion of coated granules is highly sensitive to process parameters, and achieving consistent product quality requires balancing spray rate, drying temperature, and coating solution rheology to minimize heterogeneity and maximize coating integrity.

## 3. Conclusions

This study successfully developed and optimized a humic acid–functionalized starch (St–HA) gel coating for urea-based controlled-release fertilizers using a Wurster fluidized-bed system. The integration of humic acid into the starch matrix via crosslinking with MBAA resulted in coatings with enhanced mechanical stability, improved thermal resistance, and favorable rheological properties for uniform atomization. Process optimization through CCRD demonstrated that atomizing air pressure, fluidizing gas flow rate, and spray rate collectively influenced coating integrity, surface abrasion resistance, and nutrient-release performance. Coated urea granules exhibited markedly prolonged nitrogen release in both aqueous and soil environments compared to uncoated urea, with release kinetics dominated by a combination of diffusion and polymer matrix erosion. The additional carnauba wax sealing layer further extended the release duration without compromising biodegradability. Soil burial tests confirmed significant degradation of the St–HA film, highlighting the environmental compatibility of the coating. The developed St–HA coating system provides a scalable, partially biodegradable, and bioactive solution for enhancing nitrogen-use efficiency, reducing nutrient leaching, and supporting sustainable agricultural practices.

## 4. Materials and Methods

### 4.1. Materials

Native Tapioca starch (analytical grade, moisture content < 12%) was procured from Merck KGaA, Darmstadt, Germany and used as the primary biopolymer in the coating formulation. Humic acid (technical grade, ≥90% humic substances) was obtained from Sigma-Aldrich (St. Louis, MO, USA) and used as a functionalizing agent due to its soil-conditioning and nutrient-complexing properties. The crosslinking system consisted of N,N′-methylenebisacrylamide (MBAA, ≥99%) as a bifunctional covalent crosslinker, potassium persulfate (K_2_S_2_O_8_, ≥98%) as a free-radical initiator, and sodium metabisulfite (Na_2_S_2_O_5_, ≥96%) as a redox accelerator; all three reagents were purchased from Thermo Fisher Scientific, Waltham, MA, USA. Deionized water was used as the solvent for preparing the aqueous coating solutions. For nutrient core materials, commercially available granular urea fertilizer (prill diameter 2.0–3.0 mm) was supplied by Yara International ASA (Oslo, Norway). The granules were sieved to ensure uniform particle size distribution prior to coating. For FTIR, dried coating samples were analyzed without further chemical treatment. For thermal analysis, aluminum pans and alumina crucibles were employed (NETZSCH standard).

### 4.2. Methods

#### 4.2.1. Synthesis of Coating Formulation

**Figure 11:**
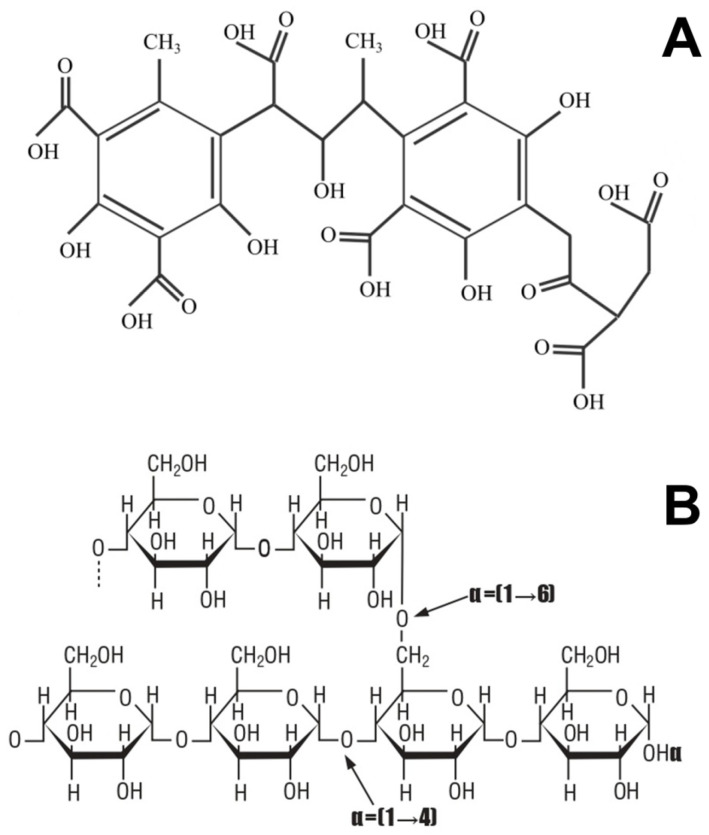
Molecular structures of (**A**) HA and (**B**) Starch.

The coating formulation was prepared by incorporating HA into a starch matrix through in situ free-radical crosslinking/graft-crosslinking, yielding a bioactive film-forming solution optimized for application via Wurster-type fluidized-bed coating. Tapioca starch (5.00 g) and humic acid (2.50 g) were dispersed in 100 mL of deionized water and heated to 70 ± 2 °C under constant magnetic stirring (600 rpm) for 30 min to ensure complete starch gelatinization and uniform dispersion of HA molecules. The mixture was then cooled to 50 ± 1 °C and purged with nitrogen gas for 10 min to eliminate dissolved oxygen and prevent premature radical quenching. The representative structures of starch and humic acid are shown in Figure 11.

Subsequently, 0.20 g of N,N′-methylenebisacrylamide (MBAA) was added to the mixture as a bifunctional crosslinker, and the solution was stirred continuously for 10 min to facilitate uniform molecular distribution and initiate preliminary network formation. Following this, 0.20 g of potassium persulfate (K_2_S_2_O_8_) was introduced as a thermal free-radical initiator, and stirring was maintained at 600 rpm for an additional 5 min. To complete the redox initiation system and enhance radical propagation, 0.20 g of sodium metabisulfite (Na_2_S_2_O_5_) was added dropwise, triggering vigorous polymerization and crosslinking reactions under maintained temperature (50 ± 1 °C) and constant stirring for 60 min.

#### 4.2.2. Characteristics of Coating Formulation

##### Rheological Analysis

The rheological behavior of the starch–humic acid-based coating formulation was evaluated using a rotational rheometer (Anton Paar MCR 302, Graz, Austria) equipped with a cone-and-plate geometry (50 mm diameter, 1° angle) and a solvent trap to minimize water evaporation. Prior to testing, the freshly prepared coating solution was equilibrated at room temperature (25 ± 1 °C) for 12 h to allow crosslinking stabilization and complete relaxation of internal stresses.

A volume of 2.0 mL of the coating solution was carefully loaded onto the rheometer plate. Excess solution was trimmed and the system was left to equilibrate for 3 min under the set temperature. A steady-state flow test was conducted over a logarithmically spaced shear rate range of 0.1 to 1000 s^−1^ to capture both low- and high-shear viscosity behavior relevant to both storage stability and pneumatic spraying in the Wurster fluidized-bed coater.

The apparent viscosity (*μ*) was recorded as a function of shear rate ($\dot{\gamma }$), and the resulting viscosity vs. shear rate profile demonstrated a clear decrease in viscosity with increasing shear rate, indicating pseudoplastic (shear-thinning) behavior typical of crosslinked hydrocolloid solutions. No yield stress was observed within the measured range.

To quantitatively describe the flow behavior, the rheological data were fitted to the Power law model using the equation:

(1) $$\mu =K\times {\dot{\gamma }}^{n-1}$$

where *μ* is the apparent viscosity (Pa·s), $\dot{\gamma }$ is the shear rate (s^−1^), *K* is the consistency index (Pa·s^n^), and *n* is the flow behavior index. The fitting was performed using non-linear regression in OriginPro 2022.

##### Fourier-Transform Infrared Spectroscopy

Fourier Transform Infrared spectroscopy was performed to investigate the functional group interactions and potential crosslinking mechanisms between starch, HA, and the crosslinker MBAA in the synthesized coating formulation. Spectra were obtained using a Nicolet iS10 FTIR spectrometer (Thermo Fisher Scientific, Waltham, MA, USA) equipped with an attenuated total reflectance (ATR) accessory and a diamond crystal.

Prior to analysis, the coating solution was dried in a vacuum oven at 50 ± 1 °C for 24 h to obtain solid films suitable for direct FTIR analysis. The dried film was then finely ground and placed in direct contact with the ATR crystal. Spectra were collected in the range of 4000–500 cm^−1^, with a spectral resolution of 4 cm^−1^, and 32 scans were averaged for each sample to improve signal-to-noise ratio.

The following samples were analyzed for comparative evaluation:

- Tapioca starch (unmodified);
- Pure humic acid;
- Physical mixture of starch and HA (without crosslinking);
- Crosslinked starch–humic acid formulation (final coating product).

All spectra were baseline-corrected and normalized prior to comparison using OMNIC software (v9.2).

##### Thermogravimetric Analysis (TGA)

Thermal behavior of the samples; including native starch, humic acid, their physical mixture, and the crosslinked starch–humic acid (St–HA) formulation, was investigated using thermogravimetric analysis (TGA) to evaluate thermal stability, decomposition patterns, and thermal transitions relevant to processing and application in fertilizer coatings.

TGA was conducted on a TGA 550 thermogravimetric analyzer (TA Instruments, New Castle, DE, USA) under a nitrogen atmosphere to prevent oxidative degradation and to ensure accurate determination of thermal decomposition behavior. Approximately 5–10 mg of each sample was weighed and placed in a platinum crucible. The samples were heated from 25 °C to 600 °C at a constant heating rate of 10 °C/min, with a nitrogen flow rate of 40 mL/min. The resulting thermograms were analyzed to determine initial weight loss temperature (onset of degradation), major decomposition stages, residual mass at 600 °C and comparative thermal stability between native components and crosslinked formulation.

The TGA characterization enabled comprehensive thermal profiling of the St–HA matrix, establishing the thermal stability range suitable for Wurster-based processing and evaluating the interaction between starch and humic acid in both physical and chemically modified states.

##### X-Ray Diffraction (XRD)

To evaluate the crystallinity and structural changes induced by chemical crosslinking, X-ray diffraction (XRD) analysis was performed on Tapioca starch, pure humic acid, their physical mixture, and the crosslinked St–HA formulation. The measurements aimed to determine the degree of crystallinity, identify peak shifts, and assess the structural interaction between starch and humic acid at the molecular level.

XRD patterns were recorded using a Bruker D8 Advance diffractometer (Bruker AXS, Karlsruhe, Germany) equipped with a Cu Kα radiation source (λ = 1.5406 Å), operating at an accelerating voltage of 40 kV and a current of 40 mA. Each finely ground and oven-dried sample was evenly spread on a sample holder to form a flat, compact surface, minimizing scattering noise. Scans were conducted over the 2θ range of 5° to 60° with a step size of 0.02° and a counting time of 1 s per step. All diffractograms were baseline corrected and normalized for comparison.

The XRD analysis was used to (i) confirm the semi-crystalline nature of native starch, (ii) evaluate the amorphous character of humic acid, (iii) observe changes in the diffraction patterns of the physical mixture versus the crosslinked formulation, and (iv) assess the degree of structural disruption or reorganization upon grafting and network formation.

##### Biodegradability Analysis of Formulation Film in Soil

To assess the environmental compatibility of the synthesized starch–humic acid (St–HA) coating formulation, the biodegradability of the crosslinked films was evaluated under simulated natural soil burial conditions. The test was designed to mimic real-world degradation behavior in an agricultural setting, where the coated urea granules would ultimately be applied.

Uniform films of the St–HA crosslinked formulation were cast by pouring the coating solution into Teflon-coated Petri dishes, followed by drying in a vacuum oven at 50 °C until a constant weight was achieved. The films were cut into square samples of known dimensions and accurately weighed (*W*_0_).

The biodegradability test was conducted following a modified version of ASTM D5988 (Standard Test Method for Determining Aerobic Biodegradation in Soil). Dried test films were buried at a depth of 5 cm in natural loamy soil (previously sieved to 2 mm and pre-characterized for pH, moisture content, and microbial activity). The soil was placed in perforated plastic containers and maintained at a constant temperature of 28 ± 2 °C and moisture content of 60% of water holding capacity, ensuring aerobic microbial activity throughout the study. Control samples (non-biodegradable polyethylene film) were used for comparison, and all tests were run in triplicate to ensure reproducibility. At regular time intervals (e.g., 7, 14, 21, and 28 days), film samples were carefully retrieved, rinsed with distilled water to remove soil particles, dried at 50 °C to constant weight, and reweighed (*W_t_*). The percentage weight loss was calculated as an indicator of biodegradation using the equation:

(2) $$Biodegradation\;\left(\%\right)=\frac{W_{0}-W_{t}}{W_{0}}\times 100$$

where *W*_0_ = Initial dry weight of the film, *W_t_* = Dry weight after burial at time *t*.

#### 4.2.3. Preparation of St–HA/Urea Coated Granules

**Figure 12:**
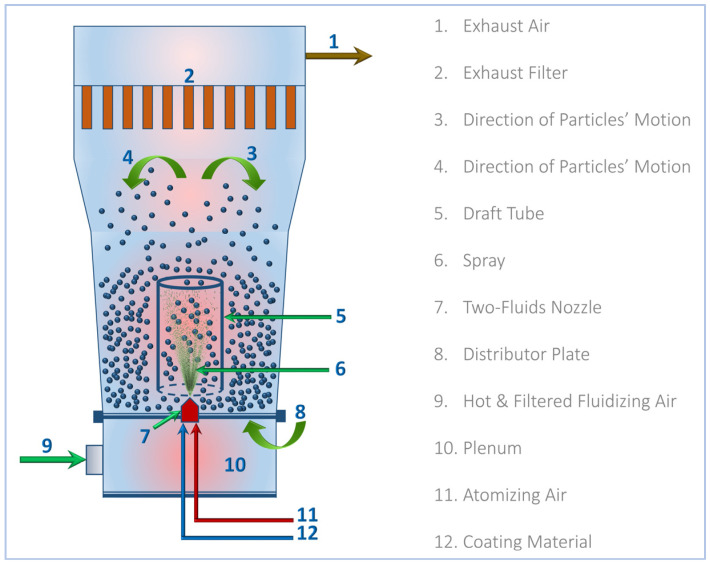
Schematic diagram for Wurster-fluidized bed coating of St–HA/Urea granules.

The starch–humic acid (St–HA) coating formulation was applied onto urea fertilizer granules using a bottom-spray Wurster-type fluidized-bed coater (GPCG-1.1, Glatt GmbH, Binzen, Germany). The schematic configuration of the equipment is shown in Figure 12. Prior to coating, the fluidizing air was drawn through a HEPA-grade filtration unit to remove particulate contaminants and then passed through an electrically controlled heating chamber, where the air was pre-heated to a desired temperature. The heated and filtered air was then introduced through the plenum chamber and distributor plate at the bottom of the Wurster column to initiate granule fluidization.

The flow rate and temperature were carefully adjusted to maintain uniform particle suspension and prevent agglomeration. Approximately 200 g of urea granules (2–3 mm) were loaded into the Wurster chamber and allowed to reach thermal equilibrium with the fluidizing air. Once stable fluidization was achieved, the St–HA coating solution (prepared fresh and maintained at the desired temperature) was introduced through a two-fluid nozzle installed centrally below the draft tube. The coating solution was atomized using compressed atomizing air and a flow rate synchronized with the solution feed rate, typically 1–2 mL/min.

The upward-moving fluidized particles entered the draft tube where they encountered the atomized droplets of the coating formulation. This configuration ensured bottom-to-top unidirectional particle motion, promoting cyclical movement and uniform layer formation on particle surfaces. The solvent in the coating formulation evaporated rapidly due to the heated air, allowing for in situ drying and film solidification during each pass. The coating process was continued for 60–90 min.

The process conditions such as atomizing air pressure, fluidizing air temperature, flowrate of coating solution and others were maintained as per the Design of Experiments (DoE) discussed in Section 4.2.5. Throughout the process, these parameters were monitored and maintained within narrow tolerances to avoid particle agglomeration or spray drying. The exhaust air passed through filters before discharge to prevent material loss and ensure environmental compliance. After coating, the St–HA/Urea granules were cooled to ambient temperature within the chamber, collected, and stored in moisture-resistant containers for further characterization.

#### 4.2.4. Characterization of St–HA/Urea Granules

##### Nutrient-Release Analysis in Water and Soil

The release behavior of nitrogen from humic acid–functionalized starch-coated urea granules (St–HA/Urea) was evaluated under two distinct conditions: (1) aqueous medium (distilled water), and (2) agricultural soil. In each case, uncoated urea granules were tested in parallel as a control to assess the effectiveness of the coating in delaying and modulating urea dissolution.

Nitrogen release in water was studied using a modified version of procedures reported by Quiceno et al. (2024), which employ UV–Vis spectrophotometric detection of ammonium ions via Nessler’s reagent [31]. A total of 5.0 g of coated urea granules and 5.0 g of uncoated urea (control) were separately placed in 250 mL Erlenmeyer flasks containing 100 mL of distilled water. The flasks were sealed with parafilm and incubated in a shaking water bath at 25 ± 1 °C and 100 rpm to ensure uniform dispersion.

At selected time intervals, 5 mL aliquots were withdrawn and replaced with equal volumes of fresh distilled water. Each aliquot was filtered through Whatman No. 42 and stored in amber vials for nitrogen analysis. Nessler’s reagent was added to each filtered sample (1 mL sample + 0.2 mL Nessler’s), and the mixture was allowed to develop color for 10 min. Absorbance was measured at 425 nm using a UV–Vis spectrophotometer (Shimadzu UV-1800, Kyoto, Japan). A standard calibration curve was constructed using known concentrations of ammonium sulfate or urea-derived ammonium nitrogen.

Cumulative nitrogen release (%) was calculated using:

(3) $$Release\;\left(\%\right)=\frac{C_{t}\cdot V+\sum C_{i}\cdot V_{i}}{M_{total\;N}}\times 100$$

where:

*C_t_*: Nitrogen concentration at time *t* (mg mL^−1^);
*V*: Volume of water (100 mL) (mL);
∑*C_i_*⋅*V_i_*: Cumulative nitrogen removed in previous aliquots (mg);
*M*_total N_: Total nitrogen content in initial 5 g urea (mg).

To simulate real agronomic conditions, nitrogen release from coated and uncoated urea granules was evaluated in natural loamy soil under aerobic conditions [32]. In total, 5.0 g of coated and uncoated urea each were placed separately in nylon mesh bags (pore size ~100 µm) and buried at a depth of 5 cm in perforated plastic pots filled with sieved (2 mm) agricultural soil. The soil moisture was adjusted to 60% of field capacity and maintained at 25 ± 2 °C throughout the study period. At scheduled intervals, mesh bags were retrieved and residual granules were carefully separated, dried, and analyzed for nitrogen content using UV–Vis after extraction with 0.1 M KCl. Residual granules recovered from the mesh bags were dried, crushed, and extracted with 0.1 M KCl under shaking for 1 h at 150–200 rpm at room temperature. The extract was filtered and analyzed by UV–Vis. Extraction was repeated until negligible additional nitrogen was detected, confirming essentially complete recovery.

##### Application of Hydrophobic Wax Overcoat in Pan Coater

To further enhance the water-resistance and prolong the nitrogen release duration, the St–HA coated urea granules were subjected to an outer coating layer of carnauba wax using a laboratory-scale pan coater (MiniCoater DRC 100, Glatt GmbH, Binzen, Germany) [33].

Prior to coating, Type 1 carnauba wax (Merck, Darmstadt, Germany), a natural vegetable wax derived from the leaves of the Brazilian carnauba palm (*Copernicia prunifera*), was weighed and melted in a stainless-steel container at 90 ± 2 °C using a thermostatically controlled oil bath. The pan coater was preheated to ~50 °C to prevent premature solidification of the wax upon contact with the granules. The inner-coated urea granules (St–HA layer) were also gently pre-warmed using warm air (40–45 °C) to improve wax adhesion and prevent thermal shock.

Approximately 350 g of pre-coated urea granules were placed into the rotating drum, which was operated at a speed of 20 rpm. The molten wax was delivered using a peristaltic pump (flow rate: 0.4 mL/min) connected to a heated stainless-steel nozzle mounted above the tumbling bed. The wax was applied continuously for a predetermined duration until the desired coating mass was achieved (e.g., 2 wt.% of the granule mass). During the coating process, warm air (60–65 °C) was supplied into the drum to maintain optimal processing temperature and ensure uniform spreading of the wax over granule surfaces.

After the wax application, the rotation was continued for an additional 10 min while a gentle stream of ambient air was introduced to accelerate solidification of the wax layer. The final coated granules were allowed to cool completely at room temperature, sieved to remove agglomerates, and stored in airtight polyethylene bags at 25 °C and 30% RH for further characterization.

##### Kinetics of Nutrient Release

To investigate the release mechanisms and quantify the kinetics of nitrogen diffusion from the coated urea granules, a comprehensive kinetic modeling study was performed based on the cumulative nitrogen release data obtained from both aqueous and soil environments [34]. The formulations tested included St–HA coated urea and St–HA/wax double-coated urea granules. The release profiles were evaluated over a 21-day period and the resulting data were normalized to 100% cumulative release to facilitate direct comparison across all formulations and media.

The zero-order model assumes a constant release rate, independent of concentration:

(4) $$M_{t}=k_{0}t$$

where *M_t_* is the amount of nutrient released at time *t*, and *k*_0_ is the zero-order release constant.

The first-order model, commonly used for systems where the release rate is concentration-dependent:

(5) $$\ln\left(1-\frac{M_{t}}{M_{\infty }}\right)=-k_{1}t$$

Here, *M*_∞_ represents the total nutrient available for release, and *k*_1_ is the first-order kinetic constant.

The Higuchi model, derived for diffusion-controlled release systems:

(6) $$M_{t}=k_{H}\sqrt{t}$$

where *k_H_* is the Higuchi dissolution constant. This model applies well to matrix-based hydrogels where nutrient diffusion is governed by Fickian mechanisms.

A more general model is the Korsmeyer–Peppas model, suitable for distinguishing between Fickian and non-Fickian transport mechanisms:

(7) $$\frac{M_{t}}{M_{\infty }}=k_{kp}t^{n}$$

In this case, *k_KP_* is the kinetic rate constant, and *n* is the release exponent. The value of *n* provides insight into the mechanism; Fickian diffusion (*n* ≤ 0.5), anomalous transport (0.5 < *n* < 1), or Case II transport (*n* = 1). Case II transport refers to a release mechanism mainly governed by polymer relaxation and matrix swelling/erosion rather than simple Fickian diffusion alone.

The Hixson–Crowell model accounts for changes in surface area and diameter of particles during release:

(8) $$M_{0}^{\frac{1}{3}}-M_{t}^{\frac{1}{3}}=k_{HC}t$$

Here, *M*_0_ is the initial amount of nutrient, and *k_HC_* is the Hixson–Crowell constant.

The model fitting was carried out via nonlinear least-squares regression using the Levenberg–Marquardt algorithm implemented in Python’s (version 3.10) scipy.optimize.curve_fit module. The initial parameter guesses were chosen based on literature-reported ranges, and model convergence was verified through residual analysis. The goodness-of-fit for each model was evaluated using the coefficient of determination (R^2^). In selected cases, root-mean-square error (RMSE) and Akaike Information Criterion (AIC) were also computed to support model discrimination (details presented in Section 2).

##### Water Retention of St–HA/Urea Granules

The water retention capacity (WRC) of the St–HA coated urea granules was assessed to determine their ability to retain moisture in a soil-like environment. This property is critical in understanding the potential of the coated fertilizer to improve water-use efficiency and reduce leaching losses in arid and semi-arid agricultural settings.

The experiment was conducted following a modified gravimetric approach based on the method reported by Esfahani et al. [35]. Briefly, 100 g of air-dried sandy loam soil was thoroughly mixed with 2.0 g of coated urea granules, and the mixture was transferred to a 300 mL plastic container perforated at the base. The soil was saturated with distilled water (excess volume) and allowed to drain freely under gravity for 24 h to reach field capacity.

After drainage, the containers were weighed (*W*_1_) and then maintained at 25 ± 2 °C in an incubator. The weight loss due to evaporation was monitored daily for 10 d by weighing the containers (*W*_2_) at the same time each day.

The retained water was calculated using the equation:

(9) $$Water\;Retention\left(\%\right)=\frac{W_{2}-W_{d}}{W_{s}}\times 100$$

where:

*W*_2_ = Weight of soil + urea + water on each day (g);
*W_d_* = Constant dry weight of soil + urea (g);
*W_s_* = Initial amount of water added at field capacity (g).

For comparison, the same procedure was conducted for (i) uncoated urea, and (ii) St–HA films without urea, to isolate the effect of both fertilizer and coating material on moisture retention behavior. All experiments were conducted in triplicate, and results were averaged. This method allowed evaluation of the ability of the biopolymer-coated granules to enhance soil moisture retention through their water-holding and slow-release properties.

##### Quantification of Surface Abrasion

Surface abrasion in coated fertilizer particles is a critical mechanical parameter, particularly during handling, packaging, and transportation, where particles are subjected to shear stresses resulting from mutual contact and friction. Assessing the extent of surface wear is essential to ensure coating integrity, as excessive abrasion can deteriorate product quality, disrupt nutrient release profiles, and diminish overall performance during storage and application.

The abrasion resistance of the coated particles was evaluated using the method outlined by Baird et al. Specifically, a known mass of coated granules was placed in a polypropylene container and subjected to mechanical agitation using a Cole-Parmer™ 8000D115 Mixer/Mill™ (Cole-Parmer Instrument Company, Vernon Hills, IL, USA) operating at 800 cycles per minute. Upon completion of the shaking process, the sample was carefully recovered and passed through a standard sieve to separate abraded fines from intact coated particles.

The degree of surface abrasion was then quantified using the following equation as reported by Baird et al. [36], providing a measure of mechanical durability under simulated handling conditions.

(10) $$\forall_{s}\left(\%\right)=100-100\left[\frac{w_{r}}{w_{i}}\right]$$

where ∀*_s_* represents surface abrasion of the coated particles, *w_r_* shows the recovered mass of the coated particles, and *w_i_* indicates the initial mass of the coated particles.

#### 4.2.5. Design of Experiments and Statistical Analysis

The design of experiments (DOE) and statistical analysis was performed using Design-Expert 13 (trial version; Stat-Ease, Minneapolis, MN, USA; downloaded from www.statease.com). A CCRD was adopted to study and optimize the coating process for St–HA–coated urea granules.

Three process variables were considered: (1) fluidized-bed temperature, °C (*X*_1_), (2) fluidizing-gas flow rate, m^3^/h (*X*_2_), atomizing-air pressure, bar (*X*_3_), and (4) spray rate, mL/s (*X*_4_). Practical lower and upper bounds for each factor were established from preliminary trial runs to ensure stable fluidization (no bed collapse or total elutriation) and continuous spraying without nozzle flooding. The factor levels used in the DOE (actual ranges and the coded levels −α, −1, 0, +1, +α) are reported in Table 4; coded variables were obtained from the actual values via:

(11) $$x_{i}=\frac{X_{i}-X_{0,i}}{∆X_{i}}$$

where *X*_0,*i*_ is the center level and Δ*X_i_* is the step size for factor *i*. For rotatability with *k* = 4 factors, the axial distance was:

(12) $$\alpha ={\left(2^{k}\right)}^{\frac{1}{4}}=2.0$$

The CCRD generated 40 experimental runs (Table 4) comprising factorial, axial, and center points. Six runs were replicated (including center-point replicates) to estimate pure error and assess reproducibility. Run order was randomized in a single block to minimize lurking time-dependent effects.

## Figures and Tables

**Table 1:** FTIR results for pure starch, humic acid, physical mixture of St-Ha, and crosslinked St–HA.

Wavenumber (cm^−1^)	Vibrational Mode	Functional Group/Assignment
**Tapioca Starch**		
~3400	Broad O–H stretching	Hydroxyl groups (–OH) involved in intra- and intermolecular hydrogen bonding
~2920	Asymmetric C–H stretching	Aliphatic –CH_2_ groups from the glucose backbone
~1630	H–O–H bending (adsorbed water)	Moisture content; water molecules hydrogen-bonded to starch
~1375	CH_2_ scissoring/bending	Methylene groups in the starch chain
~1040	C–O stretching (primary and secondary alcohols)	C–OH and C–O–C from the polysaccharide chain
~900	Skeletal vibrations of glucose ring	Indicates helical or crystalline structure of starch
~720	C–H rocking	Alkyl group (–CH_2_–) rocking vibration
**Humic Acid**		
~3400	Broad O–H stretching	Hydroxyl groups (phenolic, alcoholic, and carboxylic –OH); extensive hydrogen bonding
~2920	Asymmetric C–H stretching	Aliphatic –CH_2_/–CH_3_ groups from long-chain hydrocarbons or methyl substituents
~1710	C=O stretching	Carboxylic acid and ketonic carbonyl groups
~1610	C=C aromatic stretching/COO^−^ asymmetric	Aromatic ring vibrations and asymmetric stretching of carboxylate anions
~1260	C–O stretching	Phenolic –OH and carboxylic C–O stretching
~1020	C–O stretching/C–O–C	Alcoholic and ether groups
**St–HA Formulation (without crosslinking)**		
~3400	Broad O–H stretching	Overlapping hydroxyl groups from starch and phenolic/carboxylic OH in humic acid; enhanced hydrogen bonding may cause broadening
~2920	Asymmetric C–H stretching	Aliphatic –CH_2_/–CH_3_ groups from both starch and humic acid
~1710	C=O stretching	Carboxylic acid and ketone groups from humic acid
~1620	Aromatic C=C stretching/COO^−^ asymmetric	Aromatic rings in humic acid and COO^−^ from carboxylate anions
~1450	COO^−^ symmetric stretching/CH_2_ bending	Carboxylate groups in humic acid; bending vibrations in starch
~1125	C–O–C/C–O stretching	Ether/alcohol bands from the starch backbone
~900	C–C skeletal or ring vibrations	Starch ring vibrations; intensity retained in physical blend
**St–HA Formulation (Crosslinked)**		
~3400	Broad O–H/N–H stretching	Hydrogen-bonded –OH groups from starch and humic acid; –NH from amide (MBAA)
~2920	Asymmetric C–H stretching	Aliphatic –CH_2_/–CH_3_ from starch, humic acid, and MBAA backbone
~2850	Symmetric C–H stretching	Aliphatic –CH_2_ stretching
~1675	C=O stretching (amide I)/aromatic C=C	Amide I from MBAA crosslinker; C=O of humic acid; aromatic skeletal stretching
~1530	N–H bending/C–N stretching (amide II)	Amide II from MBAA–starch/humic linkages
~1125	C–O–C/C–O stretching	Polysaccharide ether/alcohol stretches from starch backbone

**Table 2:** Nutrient release data (recently published) from modified-starch-based films/coatings.

Ref.	Starch-Modifier	Nutrient Release Data
[1]	Polyvinyl alcohol, Kaolinite	N leaching reduced to 19.1% over 29 d; P and K leaching reduced to 48.5% and 72.3%
[2]	Polyvinyl alcohol + Fe_2_O_3_ nanoparticles	30 d cumulative leaching: N (22.87%), P (34.93%), K (84.08%)
[3]	Glycerol	Zinc release extended over 20 d from MMT/starch/glycerol composite films
[4]	Polydopamine	Extended release ~25 d
[5]	Sodium alginate + Ca^2+^ ions	~61.6% in 10 h; complete release >16 h in water; 58.5% in 25 d & >50 d for 100% release in soil
[6]	Carboxymethyl cellulose, Epichlorohydrin	84% cumulative release in 7 d
[7]	Eggshell nanoparticles	~30% release in 10 d; ~90% in 60 d
[12]	4,4-diphenylmethane diisocyanate, cellulose	100% in 35 d
[13]	Castor oil–based polyurethane + Siloxane	SSPCU7 released 100% nitrogen >63 d
[14]	Natural rubber, epoxidized NR, Glycerol	~90% N, 70% P, and 95% K released in 14 d
[15]	Sodium alginate, CaCl_2_	90% in 3–10 h
[16]	CaCl_2_	18% in 7 d in soil
[17]	Borax	76% (BAS-SRF) and 66% (ME-SRF) in water at 24 h; 94% and 92% in soil at 36 d
[18]	Acrylamide (AM) + N,N′-methylene bisacrylamide (N-MBA)	50.3% release in 15 h (aqueous), ~100% in 25 h; 46.6% in 20 d (soil); extended to 30 d in soil
[19]	Castor Oil, Hexamethylene diisocyanate	7 months
[20]	Castor oil, Polyacryl polymethylene isocyanate	60–150 days
[21]	Polyvinyl alcohol, Boric acid	51.4% N released over 90+ d; P and K included

**Table 3:** Values of diffusion exponent n based on Korsmeyer–Peppas kinetic model.

Sample	*n*	Sample	*n*	Sample	*n*	Sample	*n*
1	0.80	11	0.56	21	0.81	31	0.72
2	0.47	12	0.72	22	0.46	32	0.79
3	0.66	13	0.65	23	0.60	33	0.81
4	0.70	14	0.68	24	0.73	34	0.87
5	0.58	15	0.60	25	0.70	35	0.76
6	0.50	16	0.47	26	0.86	36	0.49
7	0.57	17	0.88	27	0.85	37	0.71
8	0.54	18	0.74	28	0.63	38	0.49
9	0.86	19	0.80	29	0.75	39	0.66
10	0.78	20	0.60	30	0.69	40	0.62

**Table 4:** Design of Experiments (In terms of actual & coded values of parameters).

Run	*X*_1_		*X*_2_		*X*_3_		*X*_4_	
	Actual	Coded	Actual	Coded	Actual	Coded	Actual	Coded
1	50	−0.6	45	−0.57	3.5	0.25	0.2	1
2	60	−0.2	50	−0.43	2	−1	0.15	0
3	45	−0.8	50	−0.43	1.5	−0.75	0.05	−1.68
4	70	0.2	65	0.00	4	1	0.25	1.68
5	65	0	75	0.29	3	0	0.1	−0.5
6	80	0.6	95	0.86	4.5	0.75	0.1	−0.5
7	90	1.68	60	−0.14	1	−1.68	0.1	−0.5
8	90	1.68	30	−1.68	3.5	0.25	0.2	1
9	75	0.4	55	−0.29	1.5	−0.75	0.25	1.68
10	70	0.2	80	0.43	4	1	0.2	1
11	55	−0.4	80	0.43	4	1	0.2	1
12	75	0.4	70	0.14	3.5	0.25	0.15	0
13	85	0.8	100	1.68	1.5	−0.75	0.05	−1.68
14	65	0	65	0.00	3	0	0.25	1.68
15	40	−1.68	80	0.43	4.5	0.75	0.15	0
16	85	0.8	50	−0.43	1.5	−0.75	0.05	−1.68
17	65	0	95	0.86	2	−1	0.2	1
18	75	0.4	70	0.14	3.5	0.25	0.15	0
19	50	−0.6	75	0.29	5	1.68	0.25	1.68
20	85	0.8	60	−0.14	1	−1.68	0.15	0
21	75	0.4	70	0.14	3.5	0.25	0.15	0
22	75	0.4	55	−0.29	3	0	0.1	−0.5
23	50	−0.6	40	−0.71	4.5	0.75	0.15	0
24	65	0	35	−0.86	2	−1	0.1	−0.5
25	40	−1.68	80	0.43	3.5	0.25	0.25	1.68
26	75	0.4	70	0.14	3.5	0.25	0.15	0
27	75	0.4	75	0.29	1	−1.68	0.15	0
28	85	0.8	100	1.68	4	1	0.15	0
29	85	0.8	85	0.57	5	1.68	0.05	−1.68
30	70	0.2	95	0.86	4.5	0.75	0.25	1.68
31	55	−0.4	45	−0.57	1.5	−0.75	0.15	0
32	75	0.4	70	0.14	3.5	0.25	0.15	0
33	65	0	65	0.00	1	−1.68	0.2	1
34	80	0.6	70	0.14	3	0	0.25	1.68
35	85	0.8	90	0.71	2.5	−0.25	0.15	0
36	85	0.8	50	−0.43	3.5	0.25	0.15	0
37	75	0.4	100	1.68	1.5	−0.75	0.2	1
38	50	−0.6	60	−0.14	1.5	−0.75	0.05	−1.68
39	65	0	70	0.14	4.5	0.75	0.25	1.68
40	90	1.68	30	−1.68	5	1.68	0.2	1

## Data Availability

The original contributions presented in this study are included in the article. Further inquiries can be directed to the corresponding author.
